# Mothers in Lockdown Due to COVID-19 in Mexico: Does Having a Paid Job Make a Difference?

**DOI:** 10.3390/ijerph191711014

**Published:** 2022-09-03

**Authors:** Nazira Calleja, Cecilia Mota

**Affiliations:** 1 Facultad de Psicología, Universidad Nacional Autónoma de México, Ciudad de México 04510, Mexico; 2 Instituto Nacional de Perinatología, Ciudad de México 11000, Mexico

**Keywords:** mothers, COVID-19, teleworking, domestic burden, coping strategies

## Abstract

Worldwide lockdowns caused by the COVID-19 pandemic had one thing in common between different countries: they highly affected family life in different ways. However, the way they affected women with young children has not been well studied. With the purpose of evaluating the experience of lockdown in Mexico in mothers with and without a paid job carried out at home, 220 Mexican women between 24 and 55 years of age, with one or more children under 15 years of age and who lived with their partner, answered online questionnaires. The results show that, although most of the domestic tasks were carried out by the mothers, the partners of those who had a paid job significantly collaborated more with them. Information and communication technologies (ICTs) were frequently used in both groups, but mothers working from home used them to a greater extent. For these mothers, work overload and confinement were among the main problems caused by the lockdown measures, while the economic situation was the main issue for the group with no paid jobs. Both groups considered family coexistence and the lack of the need to hurry as the advantages of lockdown. To face it, the participants mainly carried out coexistence and entertainment activities. To explain these differences between mothers with and without teleworking, new studies will need to be carried out.

## 1. Introduction

The lockdown measures caused by the COVID-19 pandemic around the world impacted virtually every area of daily human life. Particularly in Mexico, for mothers with young children, it meant drastic changes in domestic activities and family dynamics, which led them to generate strategies to face this new reality. However, the magnitude of these experiences could have been even greater for those who, in addition to this, had a paid job [1,2].

Traditionally, Mexican women have always overseen the upbringing and care of children, as well as the realization of domestic tasks, when it comes to family life, but nowadays, their incorporation to the labor market has increased, thus turning them into an important source of income for their families [3,4]. However, in Mexico, the COVID-19 pandemic affected both the way in which work activities were carried out and the determination of those people who preserved their jobs as well as the families that were affected by these changes [5]. In this sense, it is important to point out that, in Latin American countries with low and average incomes, the possibility of having a remunerated job during the pandemic was one of the factors associated with the impact of lockdown in terms of the emotional health and sense of well-being of the population [6].

The United Nations [7] has pointed out that women were the first responders to the COVID-19 crisis, performing the roles of caregivers, workers and volunteers. According to Del Río Lozano et al. [8] women are the ones most exposed to contagion due to the role they play as primary caregivers in both formal and informal contexts. Given this, some researchers [9,10,11,12] observed that women were not only the ones more worried about contagion but also the ones who felt more fear about their loved ones becoming sick or dying.

Particularly in some Latin American countries, it has been found that the mitigation and control measures for COVID-19, specifically the lockdown measures to keep people at home, caused an increase in domestic tasks in the care of women, [13,14] especially in terms of hygiene activities, such as laundry and the cleaning of utensils [8]. While there is evidence that these tasks where equally distributed among partners with school-age children at the beginning of the lockdown measures, a study carried out by Rodríguez et al. [15] in the United Kingdom, showed that once the shock caused by the circumstances subsided, this gender equality began to fade. In Central American countries, the pandemic brought a long list of tasks for most mothers in addition to those they usually had, including those carried out by domestic workers [16]. Moreover, mothers of children and teenagers had to pay attention to online classes and to keep an eye on the schoolwork of their children, while a more balanced distribution of the household chores was observed in those homes with no underage children [15]. According to Lacomba-Trejo et al. [17] and their study carried out in Spain in families with underage children, it was observed that the children who were confined at home due to the COVID-19 lockdown measure showed deficient strategies for emotional regulation, which generated an adverse family climate and caused emotional exhaustion in their mothers.

Regarding the mothers who had a paid job and were required to perform their work from home during the pandemic, Lemos da Costa et al. [18] found that, despite the overload of activities (work, childcare and home care), most of them indicated that the lockdown and teleworking had allowed them to be close to their children and partners. A similar effect was observed in men from middle and upper sectors in India who said the lockdown measures had allowed them to spend more time with their children [19]. Even though for some people the lockdown measures meant an opportunity to be with their families, some studies, such as the ones carried out by Farre et al. [20] and Serrano-Martínez [21] in Spanish populations, showed that mothers who worked from home found it challenging to reconcile the worlds of work and family within the same space. In fact, during the COVID-19 lockdowns, the limits in terms of shifts and working hours got frequently erased, and both family and social lives were made invisible, which caused uncertainty, frustration, emotional fatigue and mental exhaustion, [22] as well as anxiety, depression and stress [3,23,24].

In this context, the role of men at home changed as well. Research carried out with women and men who are professionally dedicated to sciences has indicated that men with this type of work participated in household chores and childcare to a much greater extent than three or four years ago, although female scientists still deal with a greater load of domestic and childcare activities [2,25] while also perceiving that this caused significant setbacks in their professional careers [19].

Furthermore, as for the modifications in daily activities and routines forced by the pandemic, the physical distancing caused by social confinement generated a digital approach by means of information and communication technologies (ICTs), particularly through the extensive use of social media, which, although already present before COVID-19, has come to occupy a preponderant place in daily family life since the first quarter of 2020 [26], keeping people in touch with family, friends and coworkers [26,27,28]. However, the forced and intensive use of technology at home could also be affecting the personal and family lives of mothers of school-age children [29], probably to a greater extent than those of mothers who have a paid job.

The adaptation to adverse circumstances is known to heavily depend on the coping mechanism of people; in this sense, there are several strategies that these mothers could have used to cope with the demands of the pandemic. For example, religion and spirituality have been associated with active coping in stressful situations generated by COVID-19 [30] and seem to minimize the adverse consequences of lockdown [31]. Other adaptive strategies that have helped people cope with the health crisis are: socializing with loved ones, exercising, focusing on study or work, avoiding negative news about COVID-19, participating in playful activities and meditating [32], as well as establishing schedules and daily routines [21].

As one of the main control and mitigation measures against SARS-CoV-2, the Mexican government established a strict lockdown that confined most of the population at home from 23 March 2020 up to 1 June 2020 when a color-coded tier system was activated to slowly ease the lockdown measures throughout the rest of the year until the present year. Independent of the different degrees and ways in which lockdowns occurred around the world, the lockdown caused by the COVID-19 pandemic may have required the mothers of young children and adolescents to not only perform traditional domestic roles but also practice new activities, such as working from home, a greater use of ICTs in their daily lives and the generation of new coping strategies. Therefore, the aim of this study was to know the way in which Mexican mothers who have a paid job and those who do not have experienced the lockdown; in particular, we sought to determine the differences between them in terms of the performance and distribution of household chores among family members (the mother, her partner, her children and someone else) and the use of ICTs. The study also analyzed their perception of problems caused by the lockdown and the possible advantages of it, as well as the strategies they used to face or alleviate its effects.

## 2. Materials and Methods

### 2.1. Participants

An invitation to participate in the study was sent via e-mail to people who attended the National Institute of Perinatology in Mexico City, an institution specializing in providing reproductive health services to the Mexican population throughout different stages of their reproductive life. They were asked to share the questionnaire on social media to increase the size of the sample, thus using snowball sampling as the main strategy. The inclusion criteria of the sample were: being a Mexican woman of legal age, having at least one child aged 15 or younger and having a partner. Out of the 353 people (97 were contacted via e-mail, and 256 received the questionnaire through their contacts) who answered the questionnaire, 33 were men, so they were discarded; and 18 of the remaining 320 were childless, which caused them to also be eliminated, thus reducing the number to 302. Out of these, 45 women who had no partner and two who had children above 15 were also excluded. Out of the 255 women who met the inclusion criteria, 145 had a paid job and 110 did not. To guarantee the measure of a similar domestic workload in both groups, the chosen 110 mothers with a paid job were those whose age, schooling, number and age of children, and number of pets in the family were not significantly different between them. The selection was made by determining the means of both groups for each variable; in those where significant differences were found, the frequencies were analyzed to detect and eliminate the cases that showed the highest and lowest values. We deemed the number of pets a necessary consideration for this selection because their presence in family life can increase the domestic burden for these women at home. The resulting sample included 220 mothers. The age of the participants ranged from 24 to 55 years with a mean of 37.90 years (SD = 6.27). More than half had bachelor degrees (56.8%). The number of children was one to five (M = 1.99, SD = 0.86). About half of the women (48.6%) had children under the age of six, and 59.1% had school-age children (6 to 11 years). In addition, 16.8% also had older children (aged 16 to 26). The partner of 76.4% of the participants had a paid job. Three out of four had one to five pets (M = 1.78, SD = 1.17). Half of the mothers had a paid job and the other half did not. Both groups did not significantly differ in any of the sociodemographic variables (Table 1).

### 2.2. Instruments

A set of questions consisting of four sections was applied:

- A sociodemographic questionnaire that explored age, education, paid work, the presence of a partner, the number and age of children, and the number of pets at home.
- A questionnaire on the use of information technologies (ICTs). It included test items that evaluated the frequent use of electronic devices (desktop computer, laptop, tablet, cellphones), social networks (WhatsApp, Facebook, Twitter, Instagram, Tik Tok) and videoconferencing applications (Skype, Hangouts, Zoom). Participants were asked to indicate how much they used each of these during lockdown (e.g., “How much have you used WhatsApp during the lockdown?”) with six options for answers that went from 1 (*I have not used it*) to 6 (*I have used it a lot*). We used these expressions and this scale as they are quite common in the daily use of Mexican Spanish.
- A questionnaire about domestic workload. It contained a list of the household chores most commonly performed in Mexican homes (shopping, cooking, washing the dishes, cleaning the kitchen, cleaning rooms, washing bathrooms, laundry and ironing, taking care of children (bathing, dressing, feeding them), doing schoolwork with children, tending to pets). It also asked participants to identify who was doing the chores during the lockdown: herself, her partner, her children and/or someone else (a maid, a family member or no one).
- A questionnaire about the problems and advantages of lockdown. One test item assessed the overall sensation experienced during lockdown: “On a scale of 1 to 10, how have you felt in this quarantine? 1 indicates that you have felt terribly bad and 10 that you have felt perfectly fine”. Two open-ended questions were included: (a) the biggest problem faced during lockdown and (b) the main perceived advantage of quarantine. Finally, a list of various strategies implemented to alleviate the effects of lockdown was presented, and participants were asked to indicate which they were using. The included activities were grouped into: cohabitation (e.g., eating together, sharing jokes and pranks); entertainment (e.g., watching TV, movies, series or YouTube videos); cognitive (e.g., reading, learning something new); physical (e.g., dancing, exercising); and reflection (e.g., focusing on the present, having a routine, praying).

### 2.3. Procedure

The questions were rendered as Google Forms questionnaires that were disseminated via e-mail and social networks (Facebook and WhatsApp) by both the researchers and the participants themselves. The initial message (see the Supplementary File) explained the context and objective of the study, which was to know the way mothers in families were experiencing the lockdown measures caused by the COVID-19 pandemic, while inviting those who met the inclusion criteria to answer the questionnaires (see the Supplementary File). The anonymity and confidentiality of the replies, which would be used only for research purposes, were expressed, and informed consent was requested. The study was approved by the Research and Ethics Committee of the National Institute of Perinatology in Mexico City. At the end of the questionnaires, the participation was appreciated, and participants were requested to share it through the same social networks. They were also told that they could withdraw their participation at any given time, in which case their data would be eliminated. The final sample included 110 with paid work and 110 without paid work; both groups did not differ in terms of their sociodemographic characteristics (age, schooling, number and age of children, pets at home). The data were collected from 7 April to 17 April 2020 during the first strict lockdown established by the Mexican government. A 10-day period for data collection was determined because the lockdown conditions were being constantly modified.

### 2.4. Data Analysis

The IBM SPSS Statistics for Windows, Version 22 (IBM Corp., Armonk, NY, USA) was used for data processing. Descriptive analyses (frequencies, percentages means and standard deviations) and comparisons with Student’s *t* tests and chi-squared tests were carried out between both groups of mothers.

## 3. Results

### 3.1. Use of Information Technologies (ICTs)

**Figure 1:**
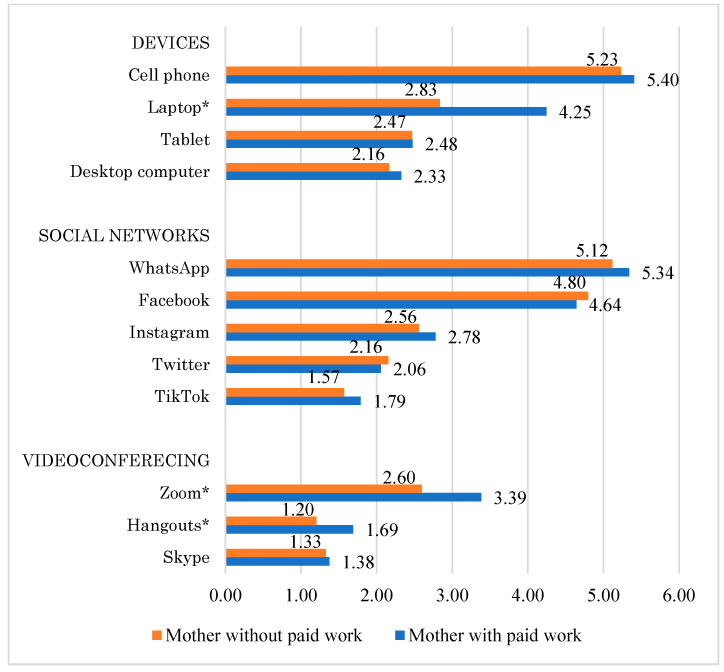
Use of ICTs by mothers with and without paid work. * *p* ≤ 0.002.

The electronic device most used by the participants was the smartphone, followed by laptops. WhatsApp and Facebook were the most used social networks, and so was Zoom in terms of videoconferencing programs (Figure 1). In three of the elements, statistically significant differences were found between mothers with and without paid work, since the former used the laptop more than the latter, *t*_(218)_ = 5.788, *p* < 0.001; Zoom, *t*_(218)_ = 3.213, *p* = 0.002; and Hangouts, *t*_(218)_ = 3.304, *p* = 0.001.

### 3.2. Household Workload

It was found that all the analyzed domestic tasks were mostly done by the mothers. Most of the activities were carried out almost entirely by them (cleaning rooms, kitchen and bathrooms, washing clothes and dishes, and caring for and doing schoolwork with their children). In terms of purchases, their partner participated in a similar proportion. Taking care of the pets seems to be the most collaborative activity as it was also carried out by their partner, children and other people at home (Table 2).

Statistically significant differences between mothers with and without paid work were only observed in one of the activities since, while 93.6% of these were those in charge of cooking, this percentage was lower (82.7%) in the participants with paid work. Although the participation percentages of the children of both groups in domestic activities were, in general, low, this was not the case with the partners of mothers with paid work, since they performed significantly more tasks than the partners of those who did not have paid work. The only activities where no significant differences between the partners of both groups were found were shopping, helping their children with homework and taking care of pets (Table 2).

### 3.3. Problems and Advantages of Lockdown

The general sensation experienced by the participants during lockdown was located in an intermediate spot between feeling “terribly bad” and “perfectly well”, considering that, on a scale of 1 to 10, the theoretical mean is 5.5 and the one obtained was 5.66 (SD = 2.54). No significant differences were found between mothers with and without paid work, *t*_(218)_ = 0.318, *p* = 0.751.

As shown in Table 3, the biggest problems faced by the participants during lockdown were diverse. The most frequently presented were the overload of activities, negative emotional states (anxiety, irritability, impatience and uncertainty) and the lockdown itself. There were significant differences between mothers with and without paid work in three of the perceived problems. A higher percentage of mothers with paid work (21.8%) than without it (9.1%) considered the overload of activities to be their main problem, *χ*^2^_(1)_ = 5.879, *p* = 0.015, as well as the confinement (17.3% versus 6.4%), *χ*^2^_(1)_ = 5.278, *p* = 0.022; while the decrease or lack of income was the most important problem for those who did not have a paid job (13.6% versus 4.5%), *χ*^2^_(1)_ = 4.455, *p* = 0.035.

Regarding the main perceived advantage of lockdown, six out of ten mothers indicated it to be the possibility of spending time with their family (children and partner). The second advantage was the fact of being unhurried and having the opportunity to rest. Only 3.6% said they did not perceive any advantage during the quarantine. No significant differences were found between mothers with and without paid work.

### 3.4. Strategies Used to Cope with Quarantine

**Figure 2:**
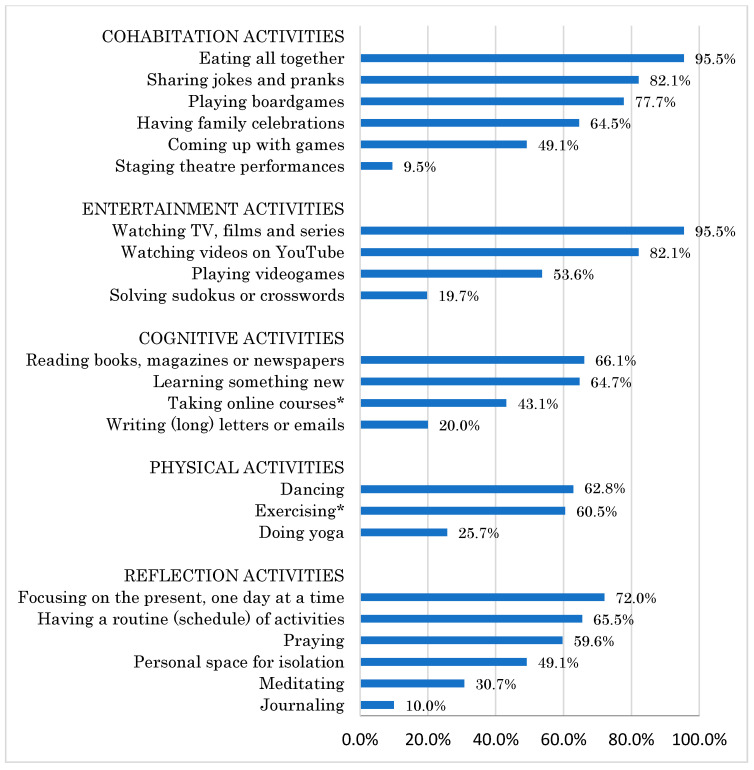
Coping strategies used by mothers during lockdown. Note: * Significant differences between participants with and without paid work, *p* < 0.05.

The strategies most used by the participants to alleviate the effects of the quarantine were related to coexistence and entertainment: eating meals together; sharing jokes and pranks; and playing board games as well as watching TV, movies, series or videos on YouTube (Figure 2). Among the cognitive activities, reading books, magazines and newspapers, and learning new content were frequently used. Dancing and exercising were the most commonly used physical activities. The most frequent reflection activities were: focusing on the present, having a fixed schedule or routine, and praying. When analyzing the strategies employed by mothers with and without paid work, statistically significant differences were found in only two of the activities: a higher percentage of mothers with paid work took online courses (51.4% versus 34.9%), *χ*^2^_(1)_ = 5.405, *p* = 0.020; and more mothers without paid work exercised (67.3% versus 53.6%), *χ*^2^_(1)_ = 3.727, *p* = 0.038.

## 4. Discussion

The aim of this research was to know if the experience of mothers of young children and adolescents during the lockdown measures imposed in Mexico due to the COVID-19 pandemic has been different for those who had a paid job and for those who did not, regarding the use of ICTs, the distribution and realization of domestic work at home, as well as in relation to the advantages and disadvantages they perceive from the lockdown they experienced and the strategies they used to face the quarantine. While the size and type of the sample limits the possible conclusions based on the collected data, these point to relevant tendencies that we highlight below. In methodological terms, it is necessary to emphasize that all participants had one or more school-age children and lived with their partner and that the mothers in both groups did not differ significantly in age, schooling, number and age of children, and number of pets. In this way, it is highly probably that the domestic burden was similar for mothers with and without paid work.

In relation to ICTs, a very frequent use of both devices and social networks and, to a lesser extent, videoconferencing applications was observed, which contrasts with what was found by other authors [24,27] in countries such as Italy and the Netherlands, where people mostly used video calls to keep in touch with their social network. The cell phone was the device most used by both groups of mothers, while those with a paid job said they used the laptop significantly more than those who did not; however, it was not possible to determine if their use of ICTs for videocalls was specifically related to either their jobs or to talk to their relatives and friends. WhatsApp and Facebook were the social networks with the highest frequency of use in both [25,28] and the Zoom videoconferencing platform was the one with the highest use, mainly by mothers with paid work. Berenguer Mayench and Brescó Biges [25] have pointed out that, in general, this platform was in the most demand for both teleworking and teaching during lockdown. ICTs have had pros and cons for mothers in lockdown. Although they allowed the realization of the school activities of the children at home, they demanded the constant accompaniment of the mothers, with a resulting cognitive and emotional overload [29,33]. In the case of mothers with paid work, ICTs made teleworking possible (a situation that a few months before seemed impossible); however, working from home required having physical spaces that, in many cases, meant invading the common living areas, causing mothers to feel uncomfortable; in addition, it meant not being able to clearly establish working hours [16,21,34]. On the other hand, devices, social networks and videoconferencing platforms also made it easier for mothers to keep in touch with their loved ones, which is a basic part of promoting mental and physical health [27,28]. Results are of interest given that the use of ICTs in mothers has been studied on very few occasions and, whenever it has occurred, it has been research-related to their use in supporting school-age children with online classes [33,35].

Regarding domestic work, the data obtained show, as expected [10,14,25] that the execution of most domestic tasks fell on the mothers, regardless of whether they had a paid job or not. However, there are some elements of interest that were observed in this study and that differ from what was reported by Castellano-Torres et al. [14]. First of all, it was a found that a greater proportion of partners of mothers with paid work carried out more house chores compared to those of mothers without it; that is to say, they prepared food, washed the dishes, cleaned the kitchen, the room and the bathrooms, and took care of the children. These results are probably the most revealing ones of the study, for they confirm that, according to the Inter-American Commission of Women [36], access to paid work is one of the factors that favors the reduction in gender inequity and allows the redistribution of activities at home and that the lockdown required by the pandemic favored the development of new masculinities both in public and private spheres [16,37,38]. Another observation was that mothers with paid work carried out some activities, such as preparing food, to a lesser extent when compared to those without it and that, in both groups, they performed some other activities, such as doing the groceries, along with their partners and with similar percentages.

Furthermore, the study demonstrated that children had little participation in the domestic tasks; mainly, they focused on cleaning their rooms and taking care of pets. It would fall on future studies to consider these findings to explore their explanation. It is worth noting that three out of every four households, on average, have two pets which, beyond the fact that they may represent an additional burden of work at home, provide psychological support to both children and adults as, according to Gómez et al. [39], they reduce the feeling of loneliness and incite the interaction of their owners with their immediate social circle, which is particularly beneficial during lockdown.

Regarding the perceived advantages and disadvantages of lockdown, our results show that the biggest problems faced by mothers and caused by this measure were the overload of activities, negative emotional states (anxiety, irritability, impatience, uncertainty), the lockdown itself, having to support and entertain their children, and the reduction in income. These findings coincide with studies that point to mothers of minor children as the most affected by work overload because children and adolescents are not yet autonomous enough to carry out many of their activities [15]. The presence of negative emotional states in mothers has also been reported in other research [40,41]. A higher proportion of mothers with paid work than without it indicated feeling the effects of overload and the lockdown itself, an expected situation given that they had to play several roles at once, while those without paid work felt more affected by economic difficulties, which is explained by the family dependence on one single income. On the other hand, the mothers of both groups considered that the lockdown gave them the particular possibility of looking after their children and spending more time with their partner, [18] of being at home with no hurries, of being able to rest, and to reflect and value what they have. The advantages of lockdown perceived by mothers reflect what is important to them: taking care of their children and their partner, slowing the rushed pace of contemporary urban life, and having the ability to reflect on what is meaningful in their lives.

Regarding the strategies they used to face the lockdown, the results show that, in general, the ones most exercised by the participants were those related to coexistence and entertainment. In agreement with Gutiérrez Cortés et al. [42], eating as a family and watching series or movies seemed to help counteract the anguish and uncertainty caused by a prolonged quarantine. Unlike the mothers who did not have a paid job (who said they exercised to a greater percentage), a higher proportion of women working from home reported performing cognitive activities, such as taking online courses. It is possible that being active in terms of work forces them to bring themselves up to date through courses and online workshops, which can also lead to a sense of well-being. It is striking that a high percentage of the participants said they resorted to reflection activities to face the situation they were going through, from focusing on the day to day and establishing a routine, to praying and meditating. Various studies have shown the fundamental role that the spiritual and religious dimension has played in subjective well-being during lockdown due to the COVID-19 pandemic [31] and have highlighted the need to consider it an effective psychological resource for coping in situations of crisis and uncertainty [43].

One of the limitations of the study was that data were only collected during the first strict lockdown meaning that the results obtained could vary at other times. Moreover, since the data were collected by electronic means, the sample comprised people who had access to technology and high educational attainment, so it is likely that in women with different socioeconomic characteristics the results may vary.

A further limitation was that although the lockdown period was explored, it is impossible to guarantee a lack of bias in the answers of the subjects.

## 5. Conclusions

The findings of the present study allowed us to observe that, in terms of the lockdown established in Mexico due to the COVID-19 pandemic, mothers with young children and adolescents who had a paid job and who had to do it from home, differed from mothers who did not have a paid job mainly because their partners shared the burden of domestic tasks with them, which allowed the distribution of the workload and contributed to a better adaptation to the conditions of lockdown. The variables that explain this fundamental difference will need to be explored in future studies. The results also show that, in general, the lockdown led to a greater use of ICTs in mothers with and without paid work, which would need to be further explored in other studies given the relevance these technologies gained in terms of the lockdown conditions during the COVID-19 pandemic. It should be noted that during the lockdown, although the mothers felt overburdened, they appreciated the possibility of living, with no hurry, with their family and that it was through entertainment, cognitive and reflection activities that they were able to face the time they spent confined in their homes in a better way.

## Figures and Tables

**Table 1:** Sociodemographic characteristics of the participants and proof of differences between mothers with and without a paid job.

Sociodemographic Variables		Total N = 220	Mothers with No Paid Job N = 110	Mothers with a Paid Job N = 110	Statistical Test
Age (in years)	Mean (SD) Range	37.90 (6.27) 24 to 55	37.68 (6.10)	38.13 (6.48)	t_(218)_ = 0.526 n.s.
Level of education	Secondary, technical career or baccalaureate (%)	27.7	33.6	21.8	χ^2^_(2)_ = 4.221 n.s.
	Bachelor degree (%)	56.8	53.6	60.0	
	Postgraduate (%)	15.5	12.7	18.2	
Number of children	Mean (SD)Range	1.99 (0.86) 1 to 5	2.08 (0.90)	1.90 (0.81)	t_(218)_ = 1.573 n.s.
With child(ren) whose age is…	Under 6 years of age (%)	48.6	52.7	44.5	χ^2^_(3)_ = 0.906 n.s.
	Ages 6 to 11 (%)	59.1	57.3	60.9	
	Ages 12 to 15 (%)	32.7	34.5	30.9	
	Ages 16 or older (%)	16.8	16.4	17.3	
Partner with a paid job (%)		76.4	71.8	80.9	χ^2^_(1)_ = 2.040 n.s.
Pets	With pets (%)	75.0	79.1	70.9	χ^2^_(1)_ = 1.552 n.s.
	Mean (SD) Range	1.78 (1.17) 1 to 5	1.86 (1.31)	1.69 (0.98)	t_(218)_ = 0.931 n.s.

**Table 2:** People in the family who carry out domestic activities and differences between mothers with and without paid work.

Domestic Activity	Mother with Paid Work	People who Carry Out Domestic Activities (%) *			
		Mother	Partner	Child(ren)	Employee Someone Else Nobody
Groceries shopping	No	62.7	60.0	3.6	2.7
	Yes	68.2	70.9	0.9	1.8
	χ^2^_(1)_ **	n.s.	n.s.	n.s.	n.s.
Cooking	No	93.6	23.6	10.0	5.5
	Yes	82.7	40.0	8.2	7.3
	χ^2^_(1)_	5.278, *p* = 0.022	6.055, *p* = 0.014	n.s.	n.s.
Doing the dishes	No	84.5	29.1	28.2	8.2
	Yes	82.7	51.8	24.5	5.5
	χ^2^_(1)_	n.s.	10.869, *p* = 0.001	n.s.	n.s.
Cleaning the kitchen	No	89.1	23.6	11.8	11.9
	Yes	83.6	44.5	15.5	9.1
	χ^2^_(1)_	n.s.	9.791, *p* = 0.002	n.s.	n.s.
Cleaning the rooms	No	97.3	31.8	38.2	9.1
	Yes	96.4	50.9	37.3	5.5
	χ^2^_(1)_	n.s.	7.496, *p* = 0.006	n.s.	n.s.
Cleaning the bathrooms	No	78.2	16.4	16.4	13.6
	Yes	74.5	35.5	10.9	11.8
	χ^2^_(1)_	n.s.	9.472, *p* = 0.002	n.s.	n.s.
Laundry and ironing	No	85.5	22.7	8.2	10.9
	Yes	82.7	37.3	8.2	10.9
	χ^2^_(1)_	n.s.	4.87, *p* = 0.027	n.s.	n.s.
Looking after the children (bathing, clothing, feeding)	No	86.4	37.3	17.3	7.3
	Yes	84.5	50.9	16.4	4.5
	χ^2^_(1)_	n.s.	4.149, *p* = 0.042	n.s.	n.s.
Helping children with homework	No	78.2	28.2	23.6	1.8
	Yes	79.1	35.5	20.0	0.9
	χ^2^_(1)_	n.s.	n.s.	n.s.	n.s.
Caring for the pet(s)	No	48.2	28.2	38.2	24.5
	Yes	49.1	33.6	30.9	29.1
	χ^2^_(1)_	n.s.	n.s.	n.s.	n.s.

* An activity can be carried out by more than one person. ** The contingency table for χ^2^ was 2 (with/without paid work), x 2 (does/does not do the activity).

**Table 3:** Percentage distribution of the participants according to the biggest problem they said they faced during lockdown and the main perceived advantage.

Biggest Problem Faced during Lockdown	Participants (%)	Main Perceived Advantage of Lockdown	Participants (%)
Activity overload *	16.1	Living with family; being present for children and/or the partner	60.5
Anxiety, irritability, impatience, uncertainty	15.5	No hurry/Rest	13.6
Confinement *	12.6	Chance to reflect and value	5.9
Homework, lessons and recreation with children	11.5	Doing enjoyable activities	5.5
Decrease or lack of income *	10.1	No advantages	3.6
Intensity of coexistence at home; overcrowding; difficulties in relationships	9.0	Savings	3.6
Fear of getting sick or that loved ones will get sick or die	5.5	Work from home (own and/or the partner)	2.7
Lack of coexistence with family and friends; isolation, loneliness	6.0	Self-care	2.3
Work stress	2.5	Other	2.3
Other	11.2		
Total	100.0	Total	100.0

* Significant differences between participants with and without paid work, *p* < 0.05.

## Data Availability

Publicly available datasets were analyzed in this study. This data can be found here: https://drive.google.com/file/d/1IBD-Bd99_VOaDqC3fNB3rtO169MhBPkg/view?usp=sharing.

## Supplementary Materials

The following supporting information can be downloaded at: https://www.mdpi.com/article/10.3390/ijerph191711014/s1.
