# Supplementary material for: Mothers in Lockdown Due to COVID-19 in Mexico: Does Having a Paid Job Make a Difference?
Source: Int J Environ Res Public Health. 2022 Sep 3;19(17):11014. doi: 10.3390/ijerph191711014 (PMC9518464; doi:10.3390/ijerph191711014)
Supplement: Supplementary file 1 [file ijerph-19-11014-s001.zip › ijerph-1792832-supplementary.pdf]

Families in lockdown due to COVID-19

Dear mothers:

Living in lockdown due to the COVID-19 pandemic has had a significant psychological impact on all of us. However, its effects are likely to be different in people of different ages and conditions. What will be the consequences for mothers? This study is focused on knowing how women who have young children (UNDER 15 YEARS OLD) have experienced lockdown and the coping strategies they have used to face it. To do this, we ask you to answer this questionnaire. We do not request any data for personal identificacion, thereby guaranteeing complete anonymity, privacy and confidentiality. Your participation is absolutely voluntary and you can leave the questionnaire unanswered at any time without sending it. This form does not involve any risk and will take you approximately 15 minutes; we ask you to answer it during a quiet moment and to share it on your social networks with other women with children under the age of 15. If you have any questions, please contact the Psychology Research Coordination via the following email: XXXX

I have read the informed consent sheet and agree to participate voluntarily*
Yes
No

In which country do you live?*
Mexico ( )
Another country ( )

If you don't live in Mexico, please specify the country where you live.

Your answer

1. Please state your gender*
Man
Woman

2. How old are you?*

Your answer

3. State your highest level of finished education*
Primary school ( )
Secondary school ( )
Baccalaureate ( )
Technical education ( )
Bachelor's degree ( )
Master's degree ( )
PhD ( )

4. Do you have children?*

If YOU DO NOT HAVE CHILDREN, this is where the survey ends for you (you do not need to submit it). Thanks.
Yes ( )
No

5. How many children do you have?*

Your answer

6. Indicate the sex of your children.

	Female	Male
Eldest child	( )	( )
Second	( )	( )
Third	( )	( )
Fourth	( )	( )

Fifth ( ) ( )

6.a Age of eldest child*

Your answer

6.b If you have a second child, indicate their age.

Your answer

6.c If you have a third child, indicate their age.

Your answer

6.d If you have a fourth child, indicate their age.

Your answer

6.d If you have a fifth child, indicate their age.

Your answer

7. Do you have a partner?*
Yes ( )
No ( )

8. Do you have a job for which you receive a salary?*
Yes ( )
No ( )

9. Does your partner have a job for which they receive a salary?*
Yes ( )
No ( )
I'm single ( )

10. Who is the breadwinner in your family?*
Only me ( )
Only my partner ( )
My partner and I ( )
Other member(s) of my family ( )

My home

11. You live in:*
House ( )
Apartment ( )

12. Your home is:*
Your own ( )
Rented ( )
Borrowed ( )
Mortgaged ( )

13. Taking into account the number of people living in your house, your home is:*
Tiny ( )
Small ( )
Regular / Adequate ( )
Big ( )
Huge ( )

14. How many bedrooms does your home have?*

Your answer

15. How many pets do you have?*
I don't have pets
1 ( )
2 ( )
3 ( )

4 ( )
More than 4 ( )

16. Does your home have a patio and/or garden?*
Yes ( )
No ( )

17. Do you have Internet at home?*
Yes ( )
No ( )

18. How many people live in your home, including you?*

Your answer

19. Have you been in contact with anyone with suspected or confirmed COVID-19?*
Yes ( )
No ( )
I don't know ( )

20. Have any of your family, friends, or close acquaintances fallen ill with COVID-19?*
Yes ( )
No ( )

21. During lockdown, have you continued receiving your salary?*
Yes, in full ( )
Yes, partial ( )
No ( )
I don't work ( )

22. In this lockdown, have you kept your job?*
Yes ( )
No ( )
I do not know yet ( )
I don't work ( )

The lockdown

23. From what date were you put under lockdown?*

In the week of March 16 to 22 ( )

In the week of March 23 to 29 Class suspensions ( )

In the week of March 30 to April 5 ( )

In the week of April 6 to 12. Easter ( )

In the week of April 13 to 19. Passover ( )

In the week of April 20 to 26. ( )

I haven't been under lockdown. ( )

24. Which is the MAIN reason why you are under lockdown?*
To protect myself ( )

To protect my family ( )

To protect others ( )

To respect the indications issued by the authorities. ( )

25. On a scale of 1 to 10, how much have you stayed at home? 1 indicates that you have gone out to do your activities outside home as you normally do and 10 that you have not left your house at all.

	1	2	3	4	5	6	7	8	9	10
I have gone out to do my activities outside home as I normally do										
I haven't left home at all.										

What activities have you done outside home during lockdown?*

26. Going to work ( )
27. Going to the market ( )
28. Going to the supermarket ( )
29. Going to the bank ( )
30. Going to the pharmacy ( )
31. Going to the bakery
32. Going to a medical appointment ( )
33. Going to a medical laboratory ( )
34. Going to a park to exercise ( )
35. Walking the dog ( )
36. Other ( )

37. On a scale from 1 to 10, how have you felt during lockdown? 1 indicates that you have felt perfectly fine and 10 that you have felt terribly bad.*

	1	2	3	4	5	6	7	8	9	10
Perfectly fine.										
Terribly bad.										

Who performs the following activities at home during lockdown? You can mark more than one. *

	Me	My partner	My children	A domestic worker	Someone else	No one does it
38. Shopping						
39. Preparing meals						
40. Doing the dishes						
41. Cleaning the kitchen						
42. Cleaning the rooms						
43. Cleaning the bathrooms						
44. Laundry and ironing						
45. Looking after the children (bathing, clothing, feeding)						

46. Taking care of pet(s)						
47. Helping children with homework						

Use of technology

How much have you used the following devices and social media during lockdown?*

	**Nothing**	**Very little**	**Little**	**Regularly**	**Much**	**A lot**
48. Desktop computer						
49. Laptop						
50. Tablet						
51. Smartphone						
52. WhatsApp						
53. Facebook						
54. Twitter						
55. Instagram						
56. TikTok						
57. Skype						
58. Hangouts						
59. Zoom						
60. Other						

During lockdown...*

	Nothing	Very little	Little	Regularly	Much	A lot
71. I have kept in touch with my relatives						
72. I have had good communication with my partner						
73. I've had fun at home with my kids						
74. I have kept in touch with my friends						
75. I have carried out activities in common with those of us who live in the house						
76. I have enjoyed spending time with my partner						
77. I have kept in touch with my relatives						
78. I have had good communication with my partner						
79. I've had fun at home with my kids						
80. I have kept in touch with my friends						
81. I have carried out activities in common with those of us who live in the house						
82. I have enjoyed spending time with my partner						

The following are strategies that people have implemented to alleviate the effects of lockdown. Which of them have you used?*

	Yes	No
83. Having a routine (schedule) of daily activities		
84. Eating together as a family		
85. Making celebrations (celebrating birthdays, small achievements or coming up with reasons to organize a party)		
86. Exercising		
87. Journaling		
88. Writing letters or e-mails (long messages)		
89. Having your own space to isolate yourself from others		
90. Watching TV, movies or series		
91. Making theatrical performances		
92. Playing board games		
93. Playing video games		
94. Coming up with games		

Coping strategies to face the lockdown (continued)

The following are strategies that people have implemented to alleviate the effects of lockdown. Which of them have you used?*

	Yes	No
95. Taking online courses		
96. Reading books, magazines or newspapers		
97. Watching YouTube videos		
98. Sharing jokes or pranks		
99. Creating a bulletin board with the events of the day		
100. Solving sudokus or crosswords		
101. Learning something new		
102. Praying		
103. Dancing		
104. Doing yoga		
105. Meditating		
106. Focusing only on the present, one day at a time		
107. Others		

108. What's the biggest problem you've faced during lockdown?*

Your answer

109. What has helped you to feel better during this lockdown?*

Your answer

110. Mention an advantage that the lockdown has given you*

Your answer
